# Impact of novel therapies for mantle cell lymphoma in the real world setting: a report from the UK's Haematological Malignancy Research Network (HMRN)

**DOI:** 10.1111/bjh.15170

**Published:** 2018-03-13

**Authors:** Alexandra Smith, Eve Roman, Simon Appleton, Debra Howell, Rod Johnson, Cathy Burton, Russell Patmore

**Affiliations:** ^1^ Epidemiology & Cancer Statistics Group Department of Health Sciences University of York York UK; ^2^ Department of Clinical Haematology St James's University Hospital Leeds UK; ^3^ Haematological Malignancy Diagnostic Service St James's University Hospital Leeds UK; ^4^ Queen's Centre for Oncology and Haematology Hull and East Yorkshire Hospitals Cottingham UK

**Keywords:** mantle cell lymphoma, international prognostic index, population‐based, novel agents

## Abstract

The treatment landscape for mantle cell lymphoma (MCL) has changed dramatically in recent years, with findings from clinical trials reporting improvements in survival. Data on the general patient population are, however, sparse; and it is unclear whether the effects observed in clinical trials have translated into the real‐world setting. To investigate this, we examined first‐line and relapsed/refractory (RR) disease management in 335 MCL patients diagnosed between 2004 and 2015 in an established population‐based patient cohort, along with data on demographic, diagnostic and prognostic factors. Marked treatment and survival changes were observed; first‐line rituximab immunotherapy, for example, increased from 32% to 86% over the 11‐year period, and median survival increased from 2·0 years among those first treated in 2004–2011 to 3·5 years among those treated in 2012–2015. Outcomes for RR disease also improved, from 8 months in 2004–2011 to 16·8 months in 2012–2015, coinciding with the introduction of agents, such as bendamustine and ibrutinib. Encouragingly, improvements were seen across all ages; 1‐year overall survival among patients over 70 years treated for RR disease almost doubled. Our analyses underscore the importance of monitoring the impact of treatment changes in the real‐world setting.

First recognized as an official entity in 1994 (Harris *et al*, 1994), mantle cell lymphoma (MCL) is a relatively rare B‐cell malignancy characterized by diverse patient pathways and a generally poor prognosis (Martin *et al*, 2017). With a median diagnostic age of around 70 years, MCL occurs 2‐3 times more frequently in men than women (Abrahamsson *et al*, 2014; Leux *et al*, 2014; Smith *et al*, 2015; Vergote *et al*, 2017); and although a small subset of patients present with localized/indolent disease that can be treated with radiotherapy and/or managed by watch and wait (W&W), the majority present with advanced disease that requires chemotherapy (McKay *et al*, 2012; Dreyling *et al*, 2017; National Comprehensive Cancer Network, 2017). Unfortunately, however, even though many patients respond well to first‐line treatment, remission is generally short‐lived, response to second and subsequent lines is poor, and survival is lower than most other lymphoma subtypes (Marcos‐Gragera *et al*, 2011; Chandran *et al*, 2012; Smith *et al*, 2015) with 5‐year overall survival estimates ranging from 30% to 50% in the general patient population (Marcos‐Gragera *et al*, 2011; Abrahamsson *et al*, 2014; Smith *et al*, 2015).

The MCL treatment landscape is, however, changing rapidly as understanding about the biology of the disease grows and the number of life‐prolonging treatments expands (Colbourn *et al*, 2016; Rule, 2016; Kahl *et al*, 2017; Martin *et al*, 2017). Encouragingly, based largely on findings from clinical trials, international guidelines for first‐line therapies are becoming increasingly systematized; dose‐intensified immunochemotherapy regimens followed by autologous stem cell transplant (ASCT) are now the standard of care for younger fitter patients, and conventional immunochemotherapy when intensive regimens are deemed inappropriate (McKay *et al*, 2012; Dreyling *et al*, 2017; Martin *et al*, 2017; National Comprehensive Cancer Network, 2017). In addition, with the aim of increasing the duration of progression‐free survival (PFS), rituximab maintenance therapy is currently the agreed standard of care for patients who respond to first‐line treatment (Kluin‐Nelemans *et al*, 2012; Vidal *et al*, 2016; Dreyling *et al*, 2017; Le Gouill *et al*, 2017; Martin *et al*, 2017). Nonetheless, although the general population of patients seem to be living longer than they did in the past, relapse remains unavoidable.

Whilst guidelines are available in the UK for first line therapy, there is no agreed standard of care for the management of patients with relapsed/refractory (RR) MCL (McKay *et al*, 2012; Zaja *et al*, 2014; Atilla *et al*, 2017; Dreyling *et al*, 2017; Martin *et al*, 2017; National Comprehensive Cancer Network, 2017); options including additional immunochemotherapy, or treatment with novel targeted agents, such as ibrutinib (Dreyling *et al*, 2016; Wang *et al*, 2016; Martin *et al*, 2017). In this context, while real‐world data has linked recent changes in first‐line management to improvements in survival for the Nordic countries and parts of France (Abrahamsson *et al*, 2011, 2014; Leux *et al*, 2014), as well as for RR MCL in Belgium(Epperla *et al*, 2017), comparable data from other countries are exceedingly sparse. Such data are, however, required in order to monitor the impact of therapeutic change at the population level, and to inform decision‐making for clinicians and regulatory agencies. This is particularly pertinent in rare diseases like MCL, where the number of patients entering clinical trials is relatively small and findings may not generalize to the patient population as a whole. To investigate the changing treatment landscape and impact on outcome in the real‐world setting, we used MCL data from an established UK population‐based patient cohort – the Haematological Malignancy Research Network (HMRN; http://www.hmrn.org).

## Methods

Data are from the UK population‐based Haematological Malignancy Research Network (HMRN; http://www.hmrn.org), which, with a catchment population of nearly 4 million people, has a socio‐demographic composition that broadly mirrors that of the UK as a whole. Initiated in 2004, full details of its structure, data collection methods and ethical approvals have been previously described (Smith *et al*, 2011, 2015). Briefly, within HMRN, patient care is provided by 14 hospitals [Teaching (academic) and District (community)], organized into five multi‐disciplinary teams (MDTs); and clinical practice adheres to national guidelines. Patients requiring an autologous stem cell transplant are treated/referred to one of the three hospitals in the Network that deliver this service. As a matter of policy, all diagnoses across the HMRN region are made to current WHO diagnostic criteria and coded by clinical specialists at a single integrated haematopathology laboratory – the Haematological Malignancy Diagnostic Service (http://www.hmds.info).

HMRN operates with Section 251 support under the NHS Act 2006, which allows full‐treatment, response and outcome data to be collected on all patients, regardless of consent, as well as ‘flagging’ for death at the national Medical Research Information Service (MRIS) and linkage to nationwide information on Hospital Episode Statistics (HES). Area‐based population counts are sourced from the Office for National Statistics. The present report includes all patients with a confirmed new diagnosis of mantle cell lymphoma (MCL) either with evidence of t(11:14) or cyclin D1 (CCND1) overexpression between 1 September 2004 and 31 August 2015; all of whom were followed up until 25 March 2017.

Analyses were conducted using standard methods in the statistical package Stata v14 (https://www.stata.com/stata14/). Time to event analyses, namely Kaplan‐Meier and Cox proportional hazard regression, were used to estimate overall survival (OS); the log rank test was used to compare survival curves. OS was assessed by demographic, prognostic and clinical characteristics including the simplified MCL International Prognostic Index (MIPI) score, which was calculated from its components (Hoster *et al*, 2008) [i.e. age at diagnosis, lactose dehydrogenase (LDH) levels, white blood cell (WBC) count and performance status] and by the cell proliferation marker Ki67 (Klapper *et al*, 2009) measured from time of diagnosis. Relative survival (RS) was also estimated, this standard approach is commonly used in population‐based studies to take into account other causes of death. The Stata program strel (v1.2.7; http://csg.lshtm.ac.uk/tools-analysis/strel-strel2/) was used to estimate relative survival (RS) and corresponding 95% Confidence Intervals (95% CI); with age and sex‐specific background mortality rates being obtained from national life tables (Cancer Research UK Cancer Survival Group, 2017, Patients still alive were censored at time of last follow‐up (25 March 2017). Outcomes for patients managed with either chemotherapy or radiotherapy were examined from the date first treatment was given.

## Results

With a median diagnostic age of 74 years (range 35–96), 339 patients were newly diagnosed with MCL during the study period; yielding a crude incidence rate of 0·9 per 100 000 per year, and the European Standard 2013 age‐standardised incidence (Eurostat 2013) rate of 1·0 per 100 000 per year. Men were almost three times more likely to be diagnosed with MCL than women; the European Standard 2013 age‐standardized (Eurostat 2013) male/female sex‐rate ratio being 2·95 (95% CI 2·30–3·78). No changes in these patterns were detectable over the 11‐year study period (2004–2015).

Around half of all patients died from their disease within three years of diagnosis; the three‐year overall and relative survival estimates being 43·9% (38·4–49·2) and 50·7% (44·4–56·7) respectively, and the corresponding one‐year estimates being 67·2% (61·8–71·9) and 70·9% (65·2–75·8). Full clinical details were available for the 335 (99%) patients who were treated within the UK National Health Service (NHS), and their baseline demographic and clinical characteristics are shown in Table 1, alongside estimates of survival. Median survival from date of diagnosis was 2·4 years; the 55 patients (16·4%) diagnosed with the particularly aggressive blastic variant having worse outcomes than those with the commoner subtype (median survivals = 1·1 and 2·8 years respectively). As expected, survival decreased with increasing age and with a median survival of 0·4 years, the outlook for the 65 patients whose performance status ranged from 2 to 4 was particularly poor [adjusted hazard ratio (HR) = 3·26 (2·35–4·53)].

**Table 1:** Median survival times and hazard ratios (95% Confidence Intervals), distributed by baseline characteristics; HMRN patients diagnosed between September 2004 and August 2015 and followed‐up until March 2017

	Number (%)	Median survival (years) (95% CI)	Hazard ratio (95% CI)	Adjusted^b^ Hazard Ratio (95% CI)
Total	335 (100)	2.4 (1.8–2.9)		
Diagnosis				
Common type	280 (83.6)	2.8 (1.9–3.4)	1	1
Blastic variant	55 (16.4)	1.1 (0.5–2.2)	1.67 (1.21–2.32)	1.96 (1.40–2.75)
Sex				
Males	223 (66.6)	2.1 (1.5–2.8)	1	1
Females	112 (33.4)	2.7 (1.9–3.5)	1.06 (0.81–1.39)	0.86 (0.65–1.13)
Age at diagnosis (years)				
Median (range)	74.0 (34.6–96.3)			
<70 years	132 (39.4)	3.9 (2.8–5.9)	0.48 (0.36–0.63)	0.41 (0.31–0.56)
≥70 years	203 (60.6)	1.6 (1.2–2.1)	1	1
Performance status (ECOG)				
0–1	266 (79.4)	3.3 (2.5–3.9)	1	1
2–4	65 (19.4)	0.4 (0.1–0.5)	3.84 (2.83–5.19)	3.26 (2.35–4.53)
Not known	4	–	–	–
B symptoms				
No	206 (61.5)	2.9 (2.1–3.8)	1	1
Yes	129 (38.5)	1.6 (0.8–2.4)	1.31 (1.01–1.70)	1.18 (0.89–1.56)
Stage				
I–II	17 (6.0)	8.6 (3.2–NR)	0.43 (0.21–0.88)	0.36 (0.17–0.75)
III–IV	264 (94.0)	2.2 (1.8–2.7)	1	1
Not fully staged^a^	54	1.5 (0.5–3.1)	1.35 (0.96–1.92)	0.67 (0.45–1.00)
Haemoglobin (g/l)				
Mean (SD)	118 (23)	–	0.88 (0.84–0.93)^c^	0.87 (0.82–0.93)^c^
β_2_‐microglobulin (mg/l)				
Mean (SD)	5.3 (3.4)	–	1.20 (1.14–1.26)^d^	1.14 (1.07–1.21)^d^
White blood cell count(10^9^/l)				
Median (p25–p75)	8.8 (6.4–15.7)	–	1.00 (1.00–1.00)^d^	1.00 (1.00–1.00)^d^
Lactate dehydrogenase				
Normal	161 (53.3)	3.9 (2.8–5.1)	1	1
Raised	141 (46.6)	1.5 (0.9–2.1)	1.81 (1.38–2.38)	1.66 (1.23–2.23)
Unknown	33	0.4 (0.1–1.4)	2.58 (1.67–3.99)	2.51 (1.58–4.00)
Simplified MIPI risk group				
Low	50 (16.7)	5.1 (3.7–9.1)	0.40 (0.26–0.62)	–
Intermediate	103 (34.3)	3.3 (2.4–4.2)	0.61 (0.45–0.83)	–
High	147 (49.0)	1.4 (0.9–2.1)	1	–
Not known	35	0.5 (0.1–1.4)	1.36 (0.89–2.08)	–
Ki67 index (%)				
<30	124 (63.3)	3.5 (2.7–4.6)	1	1
≥30	72 (36.7)	1.1 (0.8–1.9)	1.81 (1.30–2.54)	1.72 (1.05–2.80)
Not tested	139	1.8 (1.3–3.2)	1.21 (0.90–1.62)	1.00 (0.73–1.37)

95% CI: 95% confidence interval; ECOG: Eastern Cooperative Oncology Group; HMRN: Haematological Malignancy Research Network; MIPI: Mantle cell lymphoma International Prognostic Index; NR: not reached; p25–p75: 25th percentile‐75th percentile; SD: standard deviation.

a 36 computed tomography/positron emission tomography (CT/PET) staging scan only, 2 bone marrow assessment only, 16 neither bone marrow assessment nor CT/PET scan.

b Adjusted for all other variables.

c Hazard ratio for 10‐unit (g/l) increase.

d Hazard ratio for one‐unit increase.

Patients with adverse clinical and/or biological prognostic factors fared significantly less well than those without these features; the best prognosis being seen in the small group of 17 patients with Stage I and II disease, where the median survival was 8·6 years and the adjusted HR was 0·36 (0·17–0·75). With the exception of the WBC count, all blood and tumour markers (haemoglobin (Hb), β_2_‐microglobulin (β_2 _m) and LDH) were strongly associated with survival; each 10 unit (g/l) increase in Hb improving survival by 13% (HR = 0·87; 0·82–0·93) and each one unit (mg/l) increase in β_2 _m, decreasing survival by 14% (HR = 1·14; 1·07–1·21). Of the 90% of patients whose LDH was measured, 47% had elevated levels and these patients had poorer survival compared to those with normal results (adjusted HR 1·66; 1·23–2·23). The simplified MIPI score was calculated from its components; the 49% of patients who were categorised as high risk had a median survival of 1·4 years, whilst the 17% categorized as low risk had a median survival of 5·1 years. The cell proliferation marker Ki67 was measured in 168 (59%) patients at diagnosis; 72 of these (36·7%) had a proliferation of greater than 30% and a median survival of 1·1 years, compared to those with a proliferation of less than 30%, who had a median survival of 3·5 years.

### Initial management

Initial management strategies are distributed by patients’ baseline characteristics including prognostic factors and measures of disease involvement in Table 2. Whilst the majority (69%) of patients were treated with chemotherapy, only 20 (9%) went on to receive a consolidation ASCT at this point in their pathway, and these patients were, on average, the youngest (57·3 years) and fittest [Eastern Cooperative Oncology Group performance score (ECOG PS) 0/1]. A small number of patients (*n* = 9), who were less likely to have stage III/IV disease (62·5%), received radiotherapy only. The 63 (18·9%) patients managed initially by W&W were the least likely to have B‐symptoms (12·7%), and were also less likely to express high levels of Ki67 (16% ≥30%) and more likely to present with blood involvement (60·3%). Forty‐five (71·4%) of the W&W patients showed disease progression during the study period (median time to progression = 324 days); 39 of these were subsequently treated with chemotherapy and two with radiotherapy. The 18 patients who remained on W&W during the follow‐up period were more likely to have blood involvement. These patients had a very favourable outcome with a 5‐year overall survival (OS) of 81% (95% CI: 50·6–93·5) and relative survival (RS), which takes into account deaths occurring in people of the same age in the population as a whole, of 85% (47·6–96·4) (Figure S1). Thirty‐two patients were treated with a palliative approach from the outset; these patients tended to be older (median age 82·3 years) and less fit (ECOG PS>1, 68·7%).

**Table 2:** Initial management distributed by baseline characteristics; HMRN patients diagnosed between September 2004 and August 2015

	Total	Chemotherapy			Radiotherapy only	W&W			Palliative/Supportive care
		Patients	ASCT (% of those initially treated with chemotherapy)			Patients	Disease progression (% of those initially managed by W&W)		
			No	Yes			No	Yes	
Number of patients (%)	335 (100)	231 (69.0)	*211 (62.9)*	*20 (9.1)*	9 (2.7)	63 (18.9)	*18 (28.6)*	*45 (71.4)*	32 (9.6)
Male (%)	223 (66.6)	161 (69.7)	*147 (70.0)*	*13 (65.0)*	3 (33.3)	37 (58.7)	*19 (42.2)*	*26 (57.8)*	22 (68.8)
Median age, years	74.0 (34.6–96.3)	74.4	*73.9 (42.8–92.3)*	*57.3 (34.6–65.9)*	78.0 (69.6–87.1)	74.8 (46.3–95.9)	*74.4*	*75.4*	82.3 (39.4–96.3)
High MIPI Score^a^	147 (49.0)	97 (44.9)	*92 (46.9)*	*5 (25.0)*	2 (25.0)	32 (53.3)	*10 (58.8)*	*22 (51.2)*	16 (100.0)
ECOG PS 0/1 (%)	266 (79.4)	190 (82.3)	*169 (80.5)*	*20 (100)*	8 (88.9)	58 (93.5)	*16 (94.1)*	*42 (93.3)*	10 (31.3)
B‐symptoms (%)	129 (38.5)	107 (46.3)	*101 (48.1)*	*6 (30.0)*	1 (11.1)	8 (12.7)	*3 (16.7)*	*5 (11.1)*	13 (40.6)
Stage III/IV^a^ (%)	264 (94.0)	197 (94.3)	*179 (94.2)*	*18 (94.7)*	5 (62.5)	43 (95.6)	12 (85.7)	31 (100.0)	19 (100.0)
Ki67 index ≥30%^a^ (%)	72 (36.7)	57 (37.3)	*54 (38.6)*	*3 (25.0)*	3 (42.9)	4 (16.0)	*0*	*4 (18.2)*	8 (72.7)
Disease Involvement^a^									
Nodal (%)	274 (81.8)	207 (92.4)	*191 (93.2)*	*16 (84.2)*	7 (100)	37 (66.1)	*6 (37.5)*	*31 (77.5)*	23 (92.0)
Spleen (%)	171 (53.8)	132 (58.1)	*121 (58.7)*	*10 (50.0)*	2 (28.6)	22 (37.3)	*4 (25.0)*	*18 (42.9)*	15 (60.0)
Extranodal (%)	288 (92.0)	202 (91.8)	*183 (91.5)*	*19 (95.0)*	4 (50.0)	58 (96.7)	*18 (100)*	*40 (95.2)*	24 (96.0)
Blood (%)	117 (35.8)	63 (27.6)	*56 (26.9)*	*7 (36.8)*	1 (14.3)	38 (60.3)	*17 (94.4)*	*21 (46.7)*	15 (51.7)
Marrow (%)	224 (83.3)	164 (82.4)	*148 (82.2)*	*16 (84.2)*	3 (37.5)	40 (90.9)	*11 (84.6)*	*26 (83.9)*	17 (94.4)

ASCT: Autologous stem cell transplantation; ECOG PS: Eastern Cooperative Oncology Group performance score; HMRN: Haematological Malignancy Research Network; MIPI: Simplified mantle cell lymphoma International Prognostic Index; W&W: watch and wait. The italics indicate that the 211+20 are subsets of the 231 patients treated with chemotherapy. Likewise, the 18 + 45 are subsets of the 63 who were on W&W.

a Percentages calculated excluding subjects who have not been staged or tested.

### First‐line therapy

In total, 281 patients were treated either immediately after diagnosis (*n* = 240) or after an initial period of W&W (*n* = 41); 270 with chemotherapy and 11 with radiotherapy only (Table 3). Outcomes among the 157 patients treated with chemotherapy and rituximab were better than those of the 113 treated with chemotherapy alone (χ^2^ = 9·5, *P* = 0·002): the respective 1‐year and 3‐year OS estimates being 72·2% (95% CI: 64·4–78·5) and 50·3% (42·0–58·1) for those who received rituximab and 64·6% (55·0–72·7) and 29·1% (21·1–37·6) for those who did not (survival curves are presented in Figure S2). Patients who received rituximab were, however, slightly younger (median age 70·2 years) than those who did not (median 74·8 years); nonetheless the RS estimates, which take into account deaths occurring in people of the same age in the population as a whole, are broadly similar (Table 3).

**Table 3:** First‐line therapy; median age at treatment onset, overall survival (OS) and relative survival (RS): HMRN patients diagnosed between September 2004 and August 2015 and followed‐up until March 2017

	Number (%)	Median age (years)	1‐year survival (%)		3‐year survival (%)	
			Overall (95% CI)	Relative (95% CI)	Overall (95% CI)	Relative (95% CI)
Total patients	281 (100)^a^	72.2	69.4 (63.6–74.5)	72.7 (66.6–77.9)	41.8 (35.9–47.7)	47.9 (41.1–54.5)
Chemotherapy total	270 (96.1)	71.8	69.0 (63.1–74.2)	72.3 (66.0–77.6)	41.2 (35.1–47.1)	47.1 (40.1–53.7)
Chemotherapy without rituximab	113 (40.2)	74.8	64.6 (55.0–72.7)	68.2 (57.8–76.5)	29.1 (21.1–37.6)	34.4 (24.8–44.2)
Chemotherapy with rituximab	157 (56.0)	70.2	72.2 (64.4–78.5)	75.1 (66.9–81.5)	50.3 (42.0–58.1)	56.0 (46.6–64.3)
Chemotherapy + ASCT	22 (8.1)	57.4	95.5 (72.1–99.4)	96.3 (65.8–99.7)	85.5 (61.4–95.1)	87.3 (60.4–96.4)
Trial participants^b^	23 (8.5)	67.5	87.2 (65.4–95.7)	90.5 (63.5–97.9)	46.8 (25.8–65.4)	51.4 (27.7–70.8)
Radiotherapy only	11 (3.9)	76.0	80.2 (41.4–94.6)	83.9 (37.2–96.9)	59.0 (23.6–82.5)	75.5 (15.6–95.8)
Chemotherapy regimen (% of chemotherapy total, *n* = 270)						
FC‐based:	99 (35.2)	70.3	74.4 (64.6‐81.9)	76.7 (66.4‐84.3)	39.5 (29.9‐49.0)	44.2 (33.3‐54.6)
FC	54	70.5	71.7 (57.6–81.9)	74.0 (59.1–84.2)	30.2 (18.6–42.6)	34.4 (21.0–48.1)
FC‐R	45	71.2	77.6 (62.4–87.2)	79.9 (63.7–89.4)	50.7 (35.4–64.2)	55.9 (38.6 – 70.0)
CHOP +/− rituximab^c^	53 (19.6)	74.1	59.3 (44.9–71.1)	61.6 (46.4–73.7)	38.5 (25.1–51.7)	43.0 (27.8–57.3)
Chlorambucil +/− rituximab	47 (17.4)	82.6	54.9 (39.9–67.6)	59.2 (42.6–72.5)	20.8 (10.6–33.3)	26.4 (13.2–41.6)
High dose cytarabine^d^ +/− rituximab^e^	45 (16.7)	58.1	91.1 (77.9–96.5)	92.1 (77.8–97.4)	76.8 (61.0–86.8)	78.7 (62.0–88.6)
Bendamustine +/− rituximab	18 (6.7)	80.4	59.6 (33.7–78.1)	65.2 (35.1–83.9)	–	–
CVP +/− rituximab	8 (3.0)	75.9	45.4 (12.2–74.3)	46.7 (12.2–75.9)	33.8 (7.1–64.1)	35.1 (7.2–65.9)
Year of treatment						
2004–2011	182 (64.8)	71.9	68.6 (61.3–74.8)	71.7 (63.9 – 78.0)	37.9 (30.8–44.8)	43.3 (35.2–51.1)
2012–2015	99 (35.2)	73.7	70.9 (60.8–78.9)	74.7 (63.7–82.7)	50.1 (39.1–60.1)	57.8 (44.8–68.7)

95% CI: 95% confidence interval; ASCT: autologous stem cell transplantation; CHOP: cyclophosphamide, doxorubicin, vincristine, prednisolone. CVP: cyclophosphamide, vincristine, prednisolone; DHAP: dexamethasone, cytarabine, cisplatin; FC: fludarabine, cyclophosphamide; FC‐R: fludarabine, cyclophosphamide, rituximab; HyperCVAD: hyperfractionated cyclophosphamide, vincristine, doxorubicin, and dexamethasone combined with cytarabine and methotrexate; HyperCVAD/MAG: HyperCVAD + high dose methotrexate, high dose cytarabine and granulocyte colony‐stimulating factor.

a 240 were initially treated by either chemotherapy or radiotherapy and 41 patients were treated after an initial period of watch and wait.

b International Standard Randomized Controlled Trial Number (ISRCTN)81133184 (Rule *et al*, 2016).

c 1 patient also had an ASCT.

d High dose cytarabine regimens included: CHOP/DHAP, DHAP, HyperCVAD, HyperCVAD/MAG.

e 21 patients also had an ASCT.

The 22 patients who received a consolidation ASCT (two of whom were originally on W&W) were, on average, younger than those in the other groups (median age 57·4 years) and had the best outcomes; the 1‐year OS being 95·5% (72·1–99·4) and the 3‐year 85·5% (61·4–95·1), the survival curve is presented in Figure S3 (χ^2^ = 14·3, *P* = 0·0001). Only 23 (8·5%) patients were enrolled in a clinical trial; recruiting between 2002 and 2010, this trial examined the addition of rituximab to fludarabine and cyclophosphamide (FC) (Rule *et al*, 2016). As might be expected, these patients had a younger median age (67·5 years) than the chemotherapy group as a whole (70·8 years).

**Figure 1:**
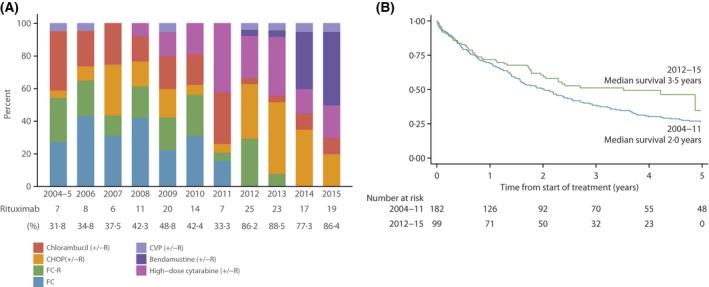
(A) First Line Regimen by Year of Treatment. (B) Overall survival by time of first line treatment. CHOP: cyclophosphamide, doxorubicin, vincristine, prednisolone; CVP: cyclophosphamide, vincristine, prednisolone; FC: fludarabine, cyclophosphamide; FC‐R: fludarabine, cyclophosphamide, rituximab; R: rituximab.

The use of rituximab immunochemotherapy increased markedly from around 30% in 2004/2005 to 86·4% in 2015, and this was accompanied by notable changes in regimen (Fig 1A). In 2004–2006, for example, patients predominantly received FC, FCR (fludarabine, cyclophosphamide, rituximab) or chlorambucil. From 2007 there was an increase in the use of CHOP‐R (cyclophosphamide, doxorubicin, vincristine, prednisolone, rituximab), and from 2008, younger fitter patients began to be treated with regimens containing high‐dose cytarabine, sometimes alongside ASCT. Bendamustine was introduced in 2012, and by 2014/2015 this drug accounted for around a third of all first‐line therapy, with fludarabine‐based regimens no longer being used.

Among treated patients, survival improved over the 11‐year study period, yielding a year‐on‐year age‐adjusted HR of 0·95% (95% CI: 0·90–0·99), and 3‐year RSs of 43·3% (35·2–51·1) and 57·8% (44·8–68·7) respectively for the periods 2004‐2011 and 2012–2015. The overall survival curves for these two time‐periods diverge a year after starting treatment (Fig 1B), with median survival increasing from 2·0 years among the 182 patients treated 2004–2011 to 3·5 years among the 99 treated 2012–2015 (χ^2^ = 4·5, *P* = 0·03). Importantly, rituximab maintenance therapy was also introduced during the study period, the first patient receiving it in 2010 and the remainder (*n* = 35) in 2012 or later; 30 of these 36 patients survived for 3 years or more after starting first‐line therapy.

**Figure 2:**
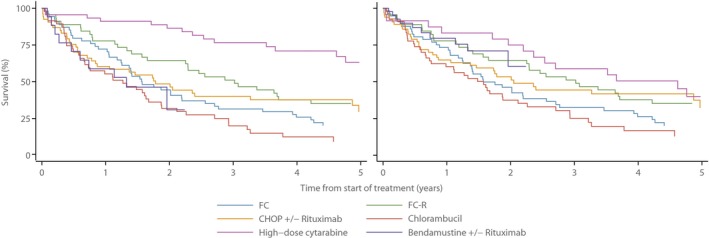
(A) Overall Survival by First Line Chemotherapy. (B) Overall Survival by First Line Chemotherapy Adjusted by Age at Treatment. CHOP: cyclophosphamide, doxorubicin, vincristine, prednisolone; FC: fludarabine, cyclophosphamide; FC‐R: fludarabine, cyclophosphamide, rituximab.

As expected, outcome varied considerably by regimen (Table 3, Fig 2A). For example, 1‐year and 3‐year OS for patients treated with high dose cytarabine (+/−rituximab) were 91·1% (77·9–96·5) and 76·8% (61·0–86·8) respectively, whereas those for chlorambucil (+/−rituximab) were 54·9% (39·9–67·6) and 20·8% (10·6–33·3). Patients treated intensively with cytarabine were, however, significantly younger (median age 58·1 years) than those receiving chlorambucil (median age 82·6 years); hence the differences, although still statistically significant, are less pronounced when age was accounted for (RS estimates in Table 3; age‐adjusted curves in Fig 2B).

### Relapse/Refractory (RR) treatment

One hundred and forty (41·8%) of the 335 patients who received first‐line treatment went on to have second‐line therapy, 45 due to refractory disease and 95 who initially responded then relapsed (median time to relapse 1·3 years, range 0·02–6·0 years). The majority of these patients received second‐line chemotherapy (127/140), with the remainder having radiotherapy (Table 4). In turn, 55 (39·3%) of the 140 patients who received second‐line treatment went on to receive third‐line therapy and so on. Overall, 233 RR treatment lines were administered (median 2 lines per patient, range 1–5) with diminishing returns (Figure S4): median survival decreasing from 0·8 years following second‐line (*n* = 140), 0·6 years following third‐line (*n* = 55), 0·4 years following fourth‐line (*n* = 28) and 0·1 years following fifth‐line (*n* = 8).

**Table 4:** Treatment for relapsed/refractory disease; median age at treatment onset, median survival and one‐year overall survival (OS); HMRN patients diagnosed between September 2004 and August 2015 and followed‐up until March 2017

	Second Line^d^				All Refractory/Relapsed Treatment Lines			
	Patients (%)	Median age (years)	Median survival (years)	1‐year overall survival (%) (95% CI)	Number (%)	Median age (years)	Median survival (years)	1‐year overall survival (%) (95% CI)
Total patients	140 (100)	72.6	0.8	45.5 (37.2–53.3)	233 (100)	72.4	0.7	39.2 (33.0–45.3)
Chemotherapy total:	127 (90.7)	71.2	0.8	44.8 (36.2–53.0)	205 (88.0)	72.0	0.7	39.9 (33.3–46.4)
without rituximab	64 (50.4)	76.9	0.7	41.9 (29.9–53.4)	104 (50.7)	74.1	0.5	31.6 (23.2–40.3)
with rituximab	63 (49.6)	68.8	1.0	47.7 (35.3–59.1)	101 (49.3)	69.3	1.0	48.5 (38.6–57.7)
Radiotherapy only	13 (9.3)	75.8	1.3	46.4 (30–61.3)	28 (12.0)	77.5	0.4	33.9 (17.5–51)
Chemotherapy regimen^a^								
FC‐based	21 (16.5)	70.7	0.8	41.8 (21.5–60.9)	30 (14.6)	73.9	0.8	44.7 (26.8‐61.0)
FC	7	83.7	0.3	24.4 (3.4–55.6)	10	81.4	0.4	28.0 (6.9–54.5)
FC‐R	14	68.2	0.8	50.4 (23.7–72.1)	20	68.2	1.0	53.4 (29.9–72.1)
CHOP(+/−R)	27 (21.3)	72.0	1.2	53.3 (33.8–69.4)	37 (18.0)	72.8	0.8	44.8 (29.2–59.3)
Chlorambucil (+/−R)	13 (10.2)	83.9	0.4	34.9 (12.2–59.1)	19 (9.3)	83.9	0.6	38.4 (17.8–58.9)
High‐dose cytarabine^b^ (+/−R)	23 (18.1)	62.2	1.1	49.8 (28.3–67.9)	38 (18.5)	62.5	0.5	31.7 (18–46.3)
Bendamustine (+/−R)	12 (9.4)	70.8	0.6	31.6 (10–56.2)	20 (9.8)	68.6	1.0	52.9 (29.4–71.8)
Ibrutinib	16 (12.6)	79.6	1.1	61.5 (33.9–80.4)	25 (12.2)	77.5	1.1	60.6 (39.5–76.4)
Other^c^	15 (11.8)	77.5	0.7	29.1 (10.8–50.5)	36 (17.6)	75.4	0.2	19.3 (9.2–32.1)
Year of treatment								
2005–2011	90 (64.3)	72.4	0.7	38.9 (29.1–48.5)	140 (60.1)	71.9	0.5	32.1 (24.8–39.6)
2012–2016	50 (25.7)	73.8	1.4	57.5 (42.9–69.7)	93 (39.9)	73.7	1.0	50.5 (39.9–60.1)

95% CI: 95% confidence interval; ASCT: autologous stem cell transplantation; CHOP: cyclophosphamide, doxorubicin, vincristine, prednisolone. CVP: cyclophosphamide, vincristine, prednisolone; DHAP: dexamethasone, cytarabine, cisplatin; FC: fludarabine, cyclophosphamide; FC‐R: fludarabine, cyclophosphamide, rituximab; HyperCVAD: hyperfractionated cyclophosphamide, vincristine, doxorubicin, and dexamethasone combined with cytarabine and methotrexate; HyperCVAD/MAG: HyperCVAD + high dose methotrexate, high dose cytarabine and granulocyte colony‐stimulating factor.

a Percentage of the total treated with chemotherapy at second line (*n* = 127) or all refractory/relapsed line (*n* = 205).

b High dose cytarabine regimens included: CHOP/DHAP, DHAP, HyperCVAD, HyperCVAD/MAG.

c Included: CVP (+/−rituximab) *n* = 6, single agent rituximab *n* = 7, bortezomib *n* = 4, etoposide *n* = 4, CHOP/bortezomib *n* = 2, thalidomide (*n* = 3), lenalidomide (*n* = 3), single agent fludarabine *n* = 3, methotrexate (it)/rituximab (*n* = 1), vincristine (*n* = 1), ofatumumab (*n* = 1).

d One patient received an allograft and two an autologous stem cell transplant.

**Figure 3:**
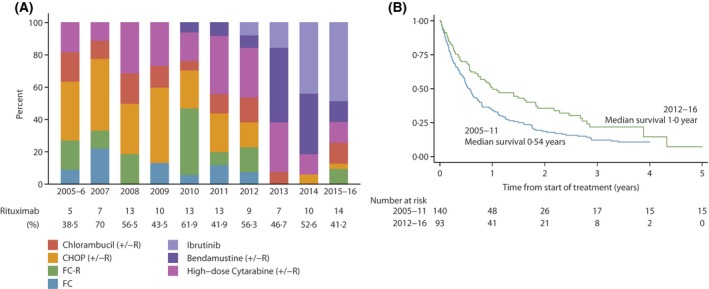
(A) Relapsed/Refractory Treatment by Year of Treatment. (B) Overall Survival by Year of Treatment, CHOP: cyclophosphamide, doxorubicin, vincristine, prednisolone; FC: fludarabine, cyclophosphamide; FC‐R: fludarabine, cyclophosphamide, rituximab; R: rituximab.

Approximately half of the patients treated for RR MCL received rituximab immunochemotherapy, but the impact was less marked than that observed at first‐line (Table 3); the 1‐years OS at second‐line was 47·7% with rituximab and 41·9% without rituximab and the corresponding figures for all lines combined were 48·5% and 31·6% respectively (Table IV). The most common RR regimens contained high‐dose cytarabine (18·5%) followed by CHOP‐R (18%) and, as with first‐line treatment, marked changes were evident over the course of the 11‐year study period (Fig 3A). However, in contrast to first‐line therapy (Fig 1A), the proportion of RR regimens delivered with rituximab showed no consistent trend over the study period (Fig 3A).

From 2012 onwards, the majority of patients with relapsed or refractory disease were treated with either bendamustine or ibrutinib, prior to this there was no dominant standard of care (Fig 3A). As with first‐line therapy, the marked changes over time in RR regimen appear to have had a positive impact on outcome, as is evident in Fig 3B (χ^2^ = 6·8, *P* = 0·009); 1‐year OS increasing from 32·1% (24·8–39·6) in 2005–2011, to 50·5% (39·9–60·1) in 2012‐2016 (Table 4). Despite the fact that ibrutinib‐treated patients were older than most of the other groups (median age 77·5 years) they had the best outcomes, with a 1‐year OS of 60·6% (39·5‐76·4) (Table 4). Although the numbers were small, stratification by age showed that the survival improvements were most marked in older patients. One‐year OS among patients aged over 70 years with RR disease, for example, increased from 28·4% (95% CI: 19·1–38·4) for those treated 2005–2011 to 53·0% (95% CI: 38·8–65·4) in those treated 2012–2016, the corresponding estimates for those under 70 years were 36·2% (95% CI: 25·2–47·3) and 47·0% (95% CI: 31·0–61·5) respectively.

## Discussion

Covering the full spectrum of MCL diagnoses, our contemporary real‐world data illustrate the underlying heterogeneity of the disease and its treatment. With a median diagnostic age of 74·0 years, our patient population is, on average, 2–5 years older than other published series (Herrmann *et al*, 2009; Abrahamsson *et al*, 2011, 2014; Epperla *et al*, 2017; Vergote *et al*, 2017). This most likely reflects the fact that all patients are identified at the point of diagnosis via a centralised diagnostic laboratory rather than retrospectively from medical records, thereby ensuring all patients are captured regardless of treatment intent; including those managed palliatively from the outset. This method of ascertainment also provides timely access to new diagnostic/prognostic measures as they are introduced into clinical practice. For example, our analyses provides the first population‐based data on the cell proliferation marker Ki67, revealing that around two‐thirds of MCL patients initially fall into the <30% category, and confirming its prognostic role alone, as well as in combination with the MIPI score (Figure S5) (Klapper *et al*, 2009; Hoster *et al*, 2016). With the exception of WBC count, all components of the MIPI predicted OS in the general patient population. The lack of association seen with WBC count may be explained by the fact the MIPI was derived using clinical trial data from cases with advanced disease (Hoster *et al*, 2008), thus excluding patients with MCL where a high WBC count may confer a prognostic benefit. Indeed, this was confirmed when the analyses were repeated separately by disease management; as WBC increased, survival increased, in those managed by W&W, whereas it fell in those treated with chemotherapy (Figure S6).

Although MCL continues to have a poor prognosis and remains one of the most challenging lymphomas to treat, our analyses confirm that the marked therapeutic changes introduced in recent years appear to be having a favourable impact on outcome in the general patient population; the median survival increasing in our cohort from 2·0 years among those who had their first treatment between 2004 and 2011 to 3·5 years among those treated between 2012 and 2015. Furthermore, significant improvements in outcome were also detectable for RR disease, with the median survival from initiation of second line treatment increasing from 8 months in 2004–2011 to 16·8 months in 2012‐2015. Importantly, however, within our cohort the benefits of therapy for RR disease declined markedly with each treatment episode, with patients receiving fifth‐line therapy, on average, surviving < 2 months.

The rarity of MCL, coupled with the range and evolving complexity of the various treatment options, can make it challenging to identify the key drivers of survival changes observed in the real‐world setting. Confirming the findings of others (Schulz *et al*, 2007; Leux *et al*, 2014), however, our data clearly demonstrate the benefit of the addition of rituximab to first‐line chemotherapy in the general patient population; at 3·1 years the median survival of those who received rituximab immunochemotherapy at first‐line was twice that of those who did not. Likewise, although patients who received rituximab immunochemotherapy for RR disease fared better than those who did not, its use did not increase during the study period, and the survival improvements seen through 2012–2015 are more likely due to the introduction of novel agents, particularly bendamustine and ibrutinib. In this context, phase II/III studies have demonstrated the effectiveness of bendamustine and ibrutinib, generally with less toxicity than that associated with other intensive chemotherapy (Rummel *et al*, 2013, 2017; Dreyling *et al*, 2016; Wang *et al*, 2016). Encouragingly, our population‐based findings confirm that these novel agents seem to be impacting particularly on the survival of patients who may be less able to withstand intensive treatment; the 1‐year OS among patients over 70 years treated for relapsed/refectory disease almost doubling, reaching 50% and matching that of patients under 70 years of age.

With the patient groups that received either a consolidation ASCT or rituximab maintenance therapy after first‐line chemotherapy both achieving 3‐year overall survivals exceeding 80%, our data also confirm the value of treatment post‐induction. Importantly, our real‐world findings for ASCT are similar to those reported in clinical trials; and a recent review of the role of ASCT in the management of MCL confirmed that this remains the standard of care for younger patients. (Dreyling & Ferrero, 2016). The proportion of patients transplanted in our study was, however, relatively small as current clinical guidelines only recommend ASCT for patients under 65 years (McKay *et al*, 2012; Dreyling *et al*, 2017) who are deemed fit enough to undergo the procedure. In our population, 34 of the 70 patients under 65 years were identified for ASCT; of these 22 had the procedure, one patient refused, and harvest attempts were unsuccessful for 10, a similar failure rate to that reported by others (Kuittinen *et al*, 2004). Indeed, the difficulty of achieving stem cell mobilisation in MCL compared to other non‐Hodgkin lymphomas has been noted by others, particularly among those previously treated with Hyper‐CVAD (hyperfractionated cyclophosphamide, vincristine, doxorubicin, and dexamethasone combined with cytarabine and methotrexate) (Hill *et al*, 2011; Kurnaz & Kaynar, 2015; Sawalha *et al*, 2018)**.**

Whilst it is well recognised that indolent MCL exists and, as with some other lymphomas, W&W in these cases is considered an acceptable management approach (Martin *et al*, 2009, 2017; Furtado & Rule, 2011), the clinical characteristics of our W&W patients support the fact that there are two distinct variants of ‘indolent’ MCL, as described in the recent revision of the 2016 WHO classification (Swerdlow *et al*, 2016; Leonard *et al*, 2017). The 18 patients whose disease did not progress during the study period were less likely to have nodal disease, but more likely to have blood involvement, and these patients had far more favourable outcomes, with 5‐year OS exceeding 80%.

Major strengths of our study include its large, well‐defined catchment population and world‐class centralised diagnostics; ensuring consistency of the diagnostic process as well as completeness of ascertainment. Accordingly, we are confident that all new MCL diagnoses were captured, which may account for the fact that our annual incidence rate [crude 0·9 per 100 000, European Standard 2013 age‐standardised (Eurostat 2013) 1·0 per 100 000] is more stable and slightly higher than that reported for some other series (Andersen *et al*, 2002; Abrahamsson *et al*, 2011, 2014; Chandran *et al*, 2012; Leux *et al*, 2014). Likewise, the fact that our cohort has a slightly higher average age and includes those treated with a palliative/supportive approach (9·6%) may also help explain why our OS is slightly lower than that reported in some other populations (Andersen *et al*, 2002; Abrahamsson *et al*, 2011, 2014; Leux *et al*, 2014). Another important factor, however, is the fact that only 40% of our patients received rituximab immunotherapy prior to 2011; this reflects national regulatory policy as rituximab has not been approved for routine use in the NHS (National Institute for Health and Care Excellence [NICE] 2016) despite being recommended for use in national guidelines in 2012 (McKay *et al*, 2012). This contrasts with the situation in some other countries, with the Nordic Lymphoma Group, for example, reporting that 50% of patients diagnosed 2000‐2005 received first‐line rituximab immunochemotherapy, increasing to 77% by 2006‐2011 and the corresponding OS estimates being 51% and 61% respectively (Abrahamsson *et al*, 2014).

The major weakness of our study, like those of many others, is small numbers. Outcomes differed by regimen, but due to the rarity of MCL the numbers of patients treated by some agents was small. Furthermore, as this is an observational study it is difficult to compare the efficacy of different regimens, as not only did the age profiles of the patients differ by therapy, but also the baseline characteristics. For example, for those treated immediately after diagnosis (i.e. not after a period of W&W), differences were seen in the demographic, diagnostic and prognostic factors by type of induction chemotherapy received (Table 5). As expected, younger and fitter patients received regimens containing high‐dose cytarabine, whereas older patients were more likely to be treated with either chlorambucil or bendamustine. Interestingly, R‐CHOP treated patients were more likely to be diagnosed with the blastic variant (31·3% vs. 19·0%) and express higher levels of Ki67 proliferation index (53·3% vs. 37·3%).

**Table 5:** First line chemotherapy by baseline characteristic

	Total	Regimen						
		FC	FC‐R	CHOP +/− Rituximab	Chlorambucil +/− Rituximab	High dose cytarabine^b^ +/− Rituximab	Bendamustine +/− Rituximab	CVP +/− Rituximab
Number of patients (%)	231	49 (21.2)	37 (16.0)	48 (20.8)	38 (16.5)	38 (16.5)	14 (6.1)	7 (3.0)
Ritxumab (%)	136 (58.9)	–	37 (100.0)	46 (95.8)	4 (10.5)	31 (81.6)	12 (85.7)	6 (85.7)
Male (%)	161 (69.7)	35 (71.4)	26 (70.3)	34 (70.8)	22 (57.9)	25 (65.8)	14 (100.0)	5 (71.4)
Median age ‐ years	74.4	69.9	71.2	74.3	81.4	57.5	81.4	76.6
High MIPI Score^a^(%)	97 (44.9)	19 (40.4)	11 (30.6)	25 (56.8)	21 (60.0)	8 (24.2)	9 (64.3)	4 (57.1)
ECOG PS 0/1^1^ (%)	190 (82.3)	39 (81.3)	34 (91.9)	38 (79.2)	26 (68.4)	38 (100.0)	11 (78.6)	4 (57.1)
B‐symptoms (%)	107 (46.3)	23 (46.9)	22 (59.5)	23 (47.9)	16 (42.1)	12 (31.6)	7 (50.0)	4 (57.1)
Stage III/IV^a^ (%)	196 (93.8)	39 (88.6)	33 (97.1)	43 (95.6)	30 (90.9)	34 (94.4)	11 (100)	6 (100)
Blastic variant^a^ (%)	44 (19.0)	9 (18.4)	5 (13.5)	15 (31.3)	4 (10.5)	7 (18.4)	1 (7.1)	3 (42.9)
Ki673 index (≥30%)^a^ (%)	57 (37.3)	11 (28.9)	10 (40.0)	16 (53.3)	6 (26.1)	8 (29.6)	3 (42.9)	3 (100.0)
Disease involvement^a^								
Nodal (%)	207 (92.4)	43 (91.5)	35 (100.0)	43 (89.6)	31 (86.1)	35 (94.6)	14 (100.0)	6 (85.7)
Spleen (%)	132 (58.1)	25 (52.1)	20 (57.1)	29 (60.4)	21 (56.8)	22 (57.9)	9 (64.3)	6 (85.7)
Extranodal (%)	202 (91.8)	40 (87.0)	32 (94.1)	40 (87.0)	34 (94.4)	37 (97.4)	13 (92.9)	6 (100)
Blood (%)	63 (27.6)	10 (20.4)	7 (20.0)	14 (29.2)	11 (28.9)	9 (24.3)	9 (64.3)	3 (42.9)
Marrow (%)	161 (80.9)	32 (76.2)	28 (80.0)	34 (81.0)	24 (80.0)	31 (86.1)	7 (87.5)	5 (83.3)
Median Year of Diagnosis	2010	2008	2009	2012	2009	2012	2014	2012

CHOP: cyclophosphamide, doxorubicin, vincristine, prednisolone. CVP: cyclophosphamide, vincristine, prednisolone; DHAP: dexamethasone, cytarabine, cisplatin; ECOG PS: Eastern Cooperative Oncology Group performance score; FC: fludarabine, cyclophosphamide; FC‐R: fludarabine, cyclophosphamide, rituximab; HyperCVAD: hyperfractionated cyclophosphamide, vincristine, doxorubicin, and dexamethasone combined with cytarabine and methotrexate; HyperCVAD/MAG: HyperCVAD + high dose methotrexate, high dose cytarabine and granulocyte colony‐stimulating factor; MIPI: Simplified mantle cell lymphoma International Prognostic Index.

a Percentages calculated excluding subjects who have not been staged or tested.

b High dose cytarabine regimens included: CHOP/DHAP, DHAP, HyperCVAD, HyperCVAD/MAG.

The development of novel agents has increased the ability to treat MCL patients who previously were unable to tolerate intensive treatments, and our findings show an improvement in survival across a population with a disease that is challenging to treat. The study highlights the importance of utilising data from high‐quality population‐based registries to monitor the impact of changes in treatment. This is especially relevant for rare diseases where it is challenging to conduct Phase 3 clinical trials (Martin, 2016), particularly in conditions where the treatment landscape is changing rapidly. We plan to continue monitoring the management and outcome of MCL, and to assess the impact of novel agents, including the introduction of immunomodulatory agents, such as lenalidomide.

## Author contributions

RP, AS, and ER were responsible for the conception and design of the study. DH and AS supervised data collection, with AS and SA undertaking the data management and statistical analyses. CB, RJ and RP provided clinical advice regarding the analysis and interpretation of the findings. All authors contributed to the final draft of the paper.

## Supporting information


**Fig S1.** Overall & relative survival for watch & wait with no disease progression (*n* = 18).
**Fig S2.** Overall survival by Ritxumab Immunochemotherapy
**Fig S3.** Overall survival by consolidation autologous stem cell transplant
**Fig S4.** Overall survival by line of treatment.
**Fig S5.** Overall Survival by biological Mantle Cell International Prognostic Index
**Fig S6.** Overall survival by mantle cell international prognostic index white blood cell count and count and first line management.
