# Supplementary material for: Impact of novel therapies for mantle cell lymphoma in the real world setting: a report from the UK's Haematological Malignancy Research Network (HMRN)
Source: Br J Haematol. 2018 Mar 13;181(2):215–28. doi: 10.1111/bjh.15170 (PMC5947165; doi:10.1111/bjh.15170)

Supplementary Figure 1 Overall & Relative Survival for Watch & Wait with no disease progression (n=18)

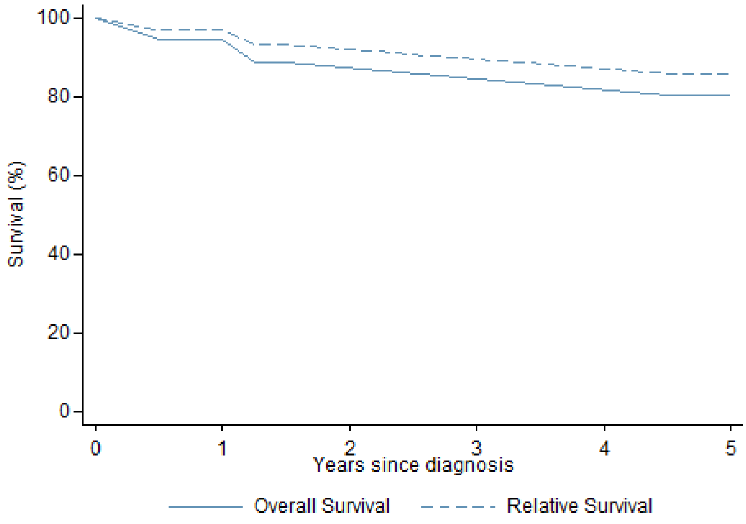

Supplementary Figure 2 Overall Survival by Ritxumab Immunochemotherapy

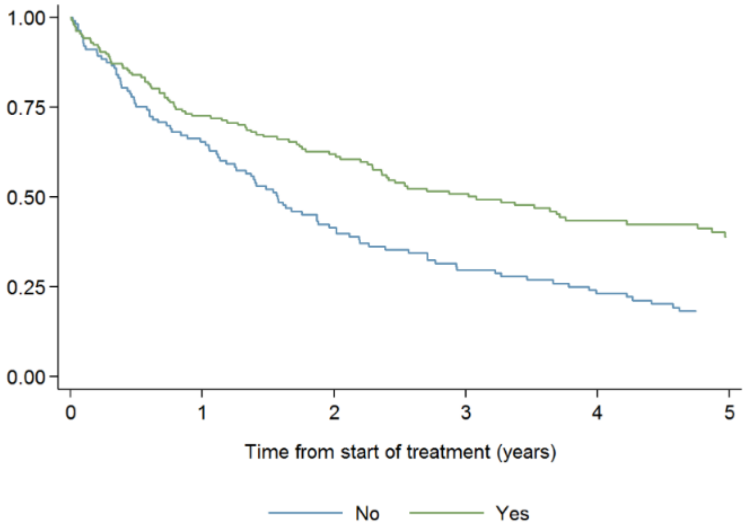

Supplementary Figure 3 Overall Survival by Consolidation Autologous Stem Cell Transplant

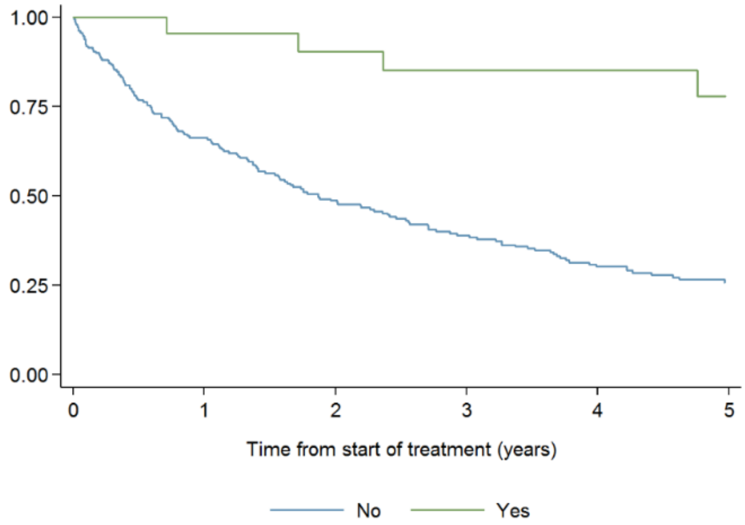

Supplementary Figure 4 Overall Survival by Line of Treatment

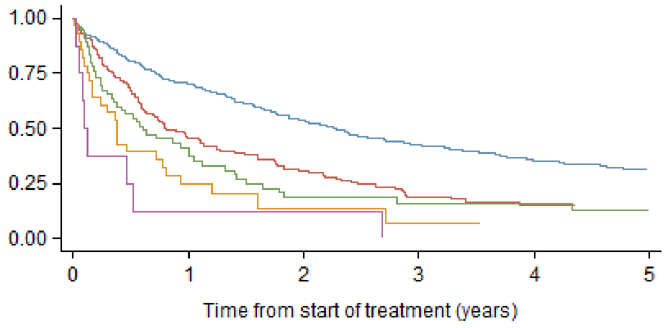

Number at risk	0	1	2	3	4	5
1st	281	197	142	102	78	48
2nd	140	61	35	18	12	11
3rd	55	20	9	6	5	4
4th	28	6	2	1	0	0
5th	8	1	1	0	0	0

1st 2nd 3rd 4th 5th

Supplementary Figure 5 Overall Survival by biological Mantle Cell International Prognostic Index

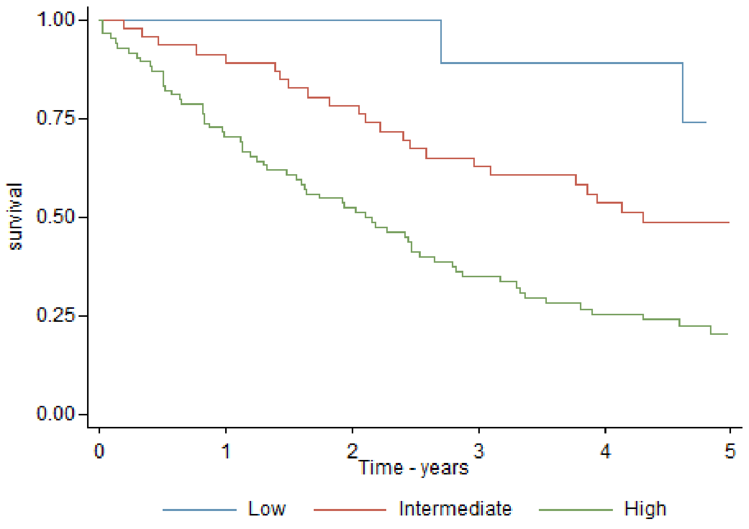

Supplementary Figure 6 Overall Survival by Mantle Cell International Prognostic Index White Blood Cell Count and First Line Management

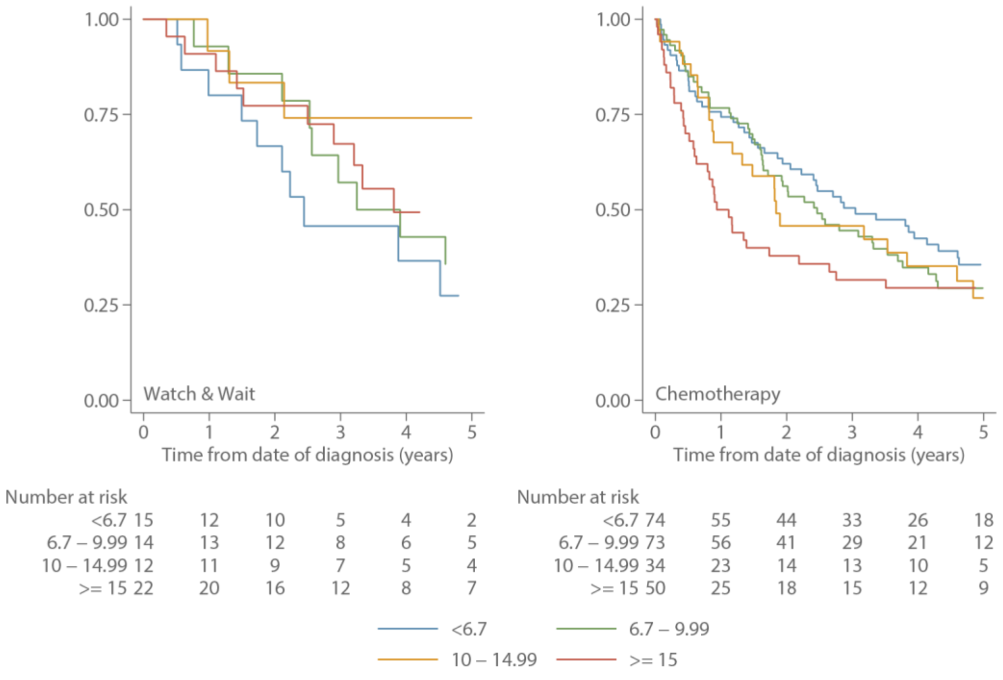

Supplement: Supplementary file 1 — Fig S1. Overall & relative survival for watch & wait with no disease progression (n = 18). Fig S2. Overall survival by Ritxumab Immunochemotherapy Fig S3. Overall survival by consolidation autologous stem cell transplant Fig S4. Overall survival by line of treatment. Fig S5. Overall Survival by biological Mantle Cell International Prognostic Index Fig S6. Overall survival by mantle cell international prognostic index white blood cell count and count and first line management. [file BJH-181-215-s001.pdf]
